# Reconciling the contrasting narratives on the environmental impact of large language models

**DOI:** 10.1038/s41598-024-76682-6

**Published:** 2024-11-01

**Authors:** Shaolei Ren, Bill Tomlinson, Rebecca W. Black, Andrew W. Torrance

**Affiliations:** 1 University of California, Riverside, USA; 2 University of California, Irvine, USA; 3 University of Kansas, Lawrence, USA; 4 Te Herenga Waka - Victoria University of Wellington, Wellington, New Zealand; 5 Massachusetts Institute of Technology, Cambridge, USA

**Keywords:** Artificial intelligence, Large language model, Environmental impact, Economic cost, Human work, Sustainability, Energy efficiency

## Abstract

The recent proliferation of large language models (LLMs) has led to divergent narratives about their environmental impacts. Some studies highlight the substantial carbon footprint of training and using LLMs, while others argue that LLMs can lead to more sustainable alternatives to current practices. We reconcile these narratives by presenting a comparative assessment of the environmental impact of LLMs vs. human labor, examining their relative efficiency across energy consumption, carbon emissions, water usage, and cost. Our findings reveal that, while LLMs have substantial environmental impacts, their relative impacts can be dramatically lower than human labor in the U.S. for the same output, with human-to-LLM ratios ranging from 40 to 150 for a typical LLM (Llama-3-70B) and from 1200 to 4400 for a lightweight LLM (Gemma-2B-it). While the human-to-LLM ratios are smaller with regard to human labor in India, these ratios are still between 3.4 and 16 for a typical LLM and between 130 and 1100 for a lightweight LLM. Despite the potential benefit of switching from humans to LLMs, economic factors may cause widespread adoption to lead to a new combination of human and LLM-driven work, rather than a simple substitution. Moreover, the growing size of LLMs may substantially increase their energy consumption and lower the human-to-LLM ratios, highlighting the need for further research to ensure the sustainability and efficiency of LLMs.

## Introduction

Large language models (LLMs) are revolutionizing many industries, enabling automated content creation^1^, improved customer service^2^, enhanced software development processes^3^, and novel approaches to the design of robots^4^. However, as LLMs become increasingly integrated into people’s daily lives and work routines, concerns have been raised about their environmental impact, particularly with regard to energy consumption, water consumption, and carbon emissions^5,6^.

Studies have shown that the training of just one LLM can consume as much energy as five cars do across their lifetimes^7^. The water footprint of AI is also substantial; for example, recent work has highlighted that water consumption associated with AI models involves data centers using millions of gallons of water per day for cooling^8^. Additionally, the energy consumption and carbon emissions of AI are projected to grow quickly in the coming years^9^, exacerbating the environmental challenges posed by this technology.

However, not all assessments of LLMs’ environmental impact are negative. One study argues that LLMs could serve as a more sustainable alternative to current work practices^10^. Others have proposed that, by automating tasks that would otherwise be performed by human workers, LLMs (or more broadly, AI systems) can provide opportunities to reduce the overall environmental footprint associated with these activities^11,12^.

These contrasting narratives, one of which positions LLMs as a “sustainability problem”, and the other of which positions them as a “sustainability solution”, underscore the need for a comprehensive assessment of the environmental impact of LLMs in comparison to humans. To address this research gap, we present a comparative life cycle assessment (LCA) of the environmental and economic costs of LLMs and human labor in the context of written content creation. While LLMs can perform various tasks, we focus on text writing as it represents a widely-used AI service and provides a specific context in which to evaluate potential efficiency gains. We acknowledge that LLM performance may vary across different content types and that our analysis does not account for qualitative differences in output between LLMs and humans. Thus, our study aims to provide a quantitative comparison of resource utilization rather than a qualitative assessment of content.

LCA is a methodology that helps quantify the environmental impacts of goods, systems, and activities across their life cycle, beginning with the sourcing of raw materials and concluding when those materials enter a waste stream^13,14^. By using an LCA approach, we seek to provide a more holistic understanding of the sustainability implications of LLM adoption. For a typical LLM, our analysis focuses on Meta’s Llama-3-70B^15^ as a representative example of LLMs and considers the task of writing a 500-word page of content. To reflect recent efficiency improvements via smaller LLMs, we also consider Gemma-2B-it as a lightweight state-of-the-art LLM. We quantify and compare the energy consumption, water consumption, carbon emissions, and economic costs associated with LLMs (Llama-3-70B/Gemma-2B-it) and human workers performing this task. Importantly, all other things being equal, the environmental impacts of LLMs are highly correlated with the model size (i.e., the number of active parameters used for inference). As such, our analysis can reveal insights into the common and relatively low environmental impacts of a variety of similar-sized LLMs beyond the ones we consider.

The results presented here focus on our best understanding of the current state of the LLM field. Although our quantitative results differ from those reported in the prior study^10^ due to variations in methodologies and our focus on conservative comparisons that lower the human-to-LLM ratios, both our findings and the prior study^10^ indicate that LLMs may serve as more efficient and cost-effective alternatives to human labor. However, the growing model sizes driven in part by the scaling law (e.g, recently released Llama-3.1-405B^16^) will likely increase the energy consumption and the associated environmental impacts of LLMs substantially. Therefore, despite the potential efficiency advantages of today’s typical and lightweight LLMs compared to human labor, we emphasize the importance of continuing and strengthening research efforts to ensure the long-term sustainability of LLMs.

**Fig. 1:**
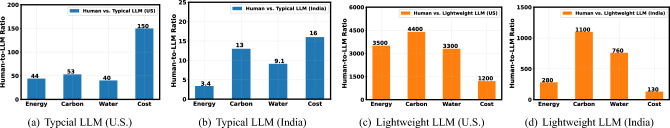
Human-to-LLM ratios in terms of the energy consumption, carbon emission, water consumption, and economic cost for writing one 500-word page of content.

## Results

The results of our comparative LCA reveal that LLMs (Llama-3-70B/Gemma-2B-it) potentially outperform a human (U.S. resident) in all four environmental and economic metrics: energy consumption (0.020/0.00024 kWh vs. 0.85 kWh per page), carbon emissions (15/0.18 grams vs. 800 grams of CO2 per page), water consumption (0.14/0.0017 liters vs. 5.7 liters per page), and economic costs ($0.08/0.01 vs. $12.1 per page), with human-to-LLM ratios ranging from 40 to 150 for Llama-3-70B and from 1,200 to 4,400 for Gemma-2B-it (with two significant figures in all the human-to-LLM ratios). We also performed a comparative LCA for the LLMs vs. an Indian resident. Although India and the U.S. have different wage levels, per capita electricity usage, carbon emissions, and water consumption, LLMs still outperform an Indian resident in all the considered metrics, resulting in human-to-LLM ratios between 3.4 and 16 for Llama-3-70B and between 130 and 1,100 for Gemma-2B-it. Despite the potential efficiency advantages of LLMs compared to human labor, we emphasize that our analysis is not intended to derail the ongoing efforts to curb LLMs’ own large environmental footprints. Instead, we recognize that these efforts must be continued and reinforced to keep LLMs sustainable in the long term, especially as the LLM size and energy consumption continue growing. In Fig. 1, we summarize our results for typical LLM vs. human (left) and lightweight LLM vs. human (right). Below, we present these results in greater detail.

### Results for typical LLM vs. human

#### Energy, carbon emission and water consumption

To estimate the environmental costs of a typical LLM, we considered Meta’s recent medium-sized Llama-3-70B model, which is one of the most powerful and widely fine-tuned open LLMs^15^. When deployed on Nvidia H100 server clusters utilizing state-of-the-practice techniques, Llama-3-70B consumes about 0.008 kWh on GPUs for producing a long output with over 350 tokens given a medium-length prompt^17^. Additionally, the non-GPU energy takes up about 30–40% of a server’s total energy on average^18^. Thus, by assuming 40% for a server’s non-GPU energy and elevating the GPU energy from 0.008 kWh to 0.010 kWh to consider a longer text output, we use an estimate of 0.017 kWh as the total server energy for Llama-3-70B to write a 500-word page of content.

The average carbon intensity for the U.S. grid was 0.39 kg/kWh in 2022, which is projected to steadily decrease in the next decade^19^. The annualized PUE, WUE for a state-of-the-art U.S. data center, and water intensity for electricity generation in the U.S. are 1.17, 0.55 L/kWh, 3.14 L/kWh, respectively^20,21^. Based on these numbers, the total operational energy consumption, operational carbon emission and water consumption for using Llama-3-70B to generate one page of content are 0.0195 kWh, 7.6 grams and 70.5 ml, respectively. While the embodied “scope-3” carbon emissions and water consumption for manufacturing GPUs and supply chains can be significant (e.g., $\sim$10–30% for servers depending on lifespans)^22–24^, official data or reliable sources for the embodied footprint are often lacking.

Here, to account for the embodied environmental footprints, we conservatively multiplied the operational environmental footprints by a factor of 2 to get the total carbon emission and water consumption by Llama-3-70B for generating a 500-word content: 15 grams and 140 ml, respectively. The conservative choice of the factor 2 is based on recent empirical studies showing the portion of embodied carbon in the overall carbon footprint for LLM inference^22,24^.

A pre-trained LLM, like the popular LLaMA model families, is used by many users and downstream tasks including customized fine-tuning to suit a variety of applications^25^. As a result, the total computational demand of LLM inference can exceed that of training by far^26^, making the amortized training cost for each LLM inference request small in real systems. Moreover, unlike the inference cost, an accurate estimate of the small amortized training cost for each inference highly depends on reliable information about the total usage of the LLM, which is lacking in the public domain. Thus, our estimate does not explicitly include the amortized training cost, which can be implicitly absorbed by our conservative choice of the factor 2 when accounting for embodied carbon and water footprints. For a fair comparison, we do not consider the amortized “training cost” (e.g., education and professional training) for performing the same task by human labor. Additionally, we neglected data centers’ carbon reduction credits obtained through a variety of programs such as Power Purchasing Agreements^27^ that may further offset the (market-based) carbon emission.

To estimate the environmental footprints of humans, we assumed that each page of content has 500 words and that the average human writing speed is 300 words/hour^10^, resulting in 1.67 hours for a human to write one page of content. While admittedly humans perform a wide variety of tasks, we amortized the human’s environmental footprints based on the number of hours needed to write one page of content. This methodology is similar in spirit to how the embodied footprints are accounted through amortization^8,22^. We excluded the environmental footprints for using auxiliary tools (e.g., a desktop or laptop) to avoid potential double counting and conduct a conservative estimate.

- *Energy.* The average U.S. per capita residential electricity consumption was 4437 kWh in 2020^28^. Thus, even excluding all the other energy usage (e.g., electricity at work and transportation), the amortized electricity usage for a human to write 500 words is about 0.85 kWh. This means that the human-to-LLM ratio for energy is 44. If we further factor in the other energy usage, the per capita primary energy consumption in the U.S. was 301 million British thermal units, or 88,200 kWh, in 2022^29^. As a result, this will increase the human-to-LLM ratio to $\sim$860. We used the more conservative ratio of 44. The per capita electricity consumption in India was 1395 kWh in fiscal year 2024, with the residential sector accounting for 25% (or 349 kWh)^28^. As a result, the human-to-LLM ratio is 3.4 in India even though, for a conservative estimate, we only considered the amortized residential electricity consumption.
- *Carbon emission.* The average U.S. per capita electric power-related carbon emission was 4.20 metric tons in 2023. Even without considering carbon emissions for other categories (e.g., transportation), the amortized carbon emission for an average U.S. person to write one page of content is about 800 grams, resulting in a human-to-LLM ratio of 53. If we factor in all carbon emissions, the average US per capita carbon emission is about 14.1 metric tons per year^30^. Thus, the amortized carbon emission for a US person to write one page of content is about 2,700 grams, resulting in a human-to-LLM ratio of $\sim$177. In this paper, we use the more conservative estimate of 53. Based on India’s average carbon intensity for electricity generation of 0.716 kg/kWh, the per capita electric power-related carbon emission in India was about 0.999 metric tons in 2023^31^, resulting in an estimated human-to-LLM carbon ratio of 13.
- *Water consumption.* Water consumption refers to the amount of evaporated water that does not return to the original source^8^, and is sometimes considered “permanently lost” while it enters the global water cycle^32^. The U.S. per capita water withdrawal at home was 309.96 L/day^33^. We used 10% and 30% for the consumptive rates for urban households and rural households, respectively^34^. As of 2020, 20% of the US population was rural, while the remaining 80% was urban/suburban^35^. Thus, this yields an average water consumptive rate of 14%, leading to the U.S. per capita water consumption at home of 43.40 L/day. For fair comparison, we included the indirect water consumption 2.67 L for generating 0.85 kWh of electricity used by a human to write a 500-word page of content. Thus, even without considering the human water consumption at workplaces, the amortized water consumption for a human to write one page of content is about 5.68L, resulting in a human-to-LLM ratio of 40. In India, the benchmarks for per capita water withdrawal in urban areas and rural areas are 135 L/day and 55 L/day, respectively^36^. India’s population is 65% rural and 35% urban with consumptive rates of 30% and 10%, respectively. Thus, we estimated India’s overall per capita water consumption at about 15 L/day. By considering the amortized electricity consumption of 0.066 kWh by an Indian person to perform the writing task and 3.4 L/kWh for electricity generation in India, we obtained an indirect water consumption of 0.22 L associated with electricity usage. This gives the total amortized water consumption of 1.3 L for an Indian person to perform the writing task, leading to a human-to-LLM ratio of 9.1 in India.

#### Economic costs

As Llama-3-70B is a free LLM, we considered the cost of using ChatGPT as a substitute for text generation in our economic analysis. While OpenAI offers a variety of LLMs at different prices, we considered the most expensive option as of May 2024 for a conservative estimate: $120.00 per 1M output tokens for GPT-4-32k^37^, where “$” means U.S. dollars in this paper. We excluded the tokens for input. To generate a 500-word page of content, we need about 667 tokens, which costs about $0.08.

We used the U.S. federal minimum wage (for covered nonexempt employees) to estimate human costs for performing the same task. Excluding fringe benefits and other applicable costs, the federal minimum wage is $7.25/hour^38^. Thus, assuming a writing speed of 300 words/hour, the minimum economic cost for a human to write a 500-word page of content is $12.08, resulting in a human-to-LLM cost ratio of 150. In India, the Chief Labour Commissioner (Central) provides guidelines for sector-specific daily minimum wages, which are mostly higher than $6 each day^39^. By the assumption of eight working hours each day, we obtain a human-to-LLM cost ratio of 16, suggesting that LLMs could even be more cost-effective than human labor for writing tasks in developing countries with relatively lower wages.

### Results for lightweight LLM vs. human

The recent efforts in efficiency improvement such as model compression have substantially reduced LLM’s energy usage. To reflect this trend, we considered Gemma-2B-it, a lightweight state-of-the-art open LLM that can be readily deployed on a single GPU or even modern mobile devices^40^. Our measurement on a single Nvidia A100 GPU over 5,000 testing prompts without optimal batching showed that Gemma-2B-it consumes about $\sim$0.0002 kWh for producing a 500-word response, roughly 1% energy of Llama-3-70B for doing the same job. The 0.0002 kWh energy includes the RAM and CPU energy consumption, but excludes the cooling overhead in data centers. In our estimate, we used 0.0002 kWh to represent the energy consumption for a lightweight LLM, although this value could be lower when further using advanced hardware and software optimization techniques^17^. By assuming the same deployment environment in a state-of-the-art U.S. data centers, the energy consumption, carbon emission and water consumption for Gemma-2B-it to write a 500-word page of content are 0.00024 kWh, 0.18 gram and 1.69 ml, respectively. Therefore, this results in the following human-to-LLM ratios: 3500 (energy), 4400 (carbon), and 3300 (water). In India, these ratios are 280 (energy), 1100 (carbon), and 760 (water), respectively.

In addition to the expensive option of GPT-4-32k, OpenAI offers multiple alternative LLMs for ChatGPT with lower prices. While the cheapest option GPT-3.5-Turbo only costs $2.00 per 1M output tokens, we considered the recently released GPT-4o offered by OpenAI at $15.00 per 1M output tokens as of May 2024^37^. Therefore, the human-to-LLM cost ratio is 1200 in the U.S. and 130 in India.

## Discussion

### Justification of comparison methodology

Comparing the environmental impact of LLMs to an amortized portion of a human’s total footprint may seem unconventional, but we believe it is necessary for accurately assessing environmental costs in labor and production.

When a company employs someone, they are not just paying for task-specific energy expenditure. They are effectively renting a portion of that person’s life — typically 40 hours per week. During this time, all of the individual’s environmental impacts, from commuting to basic life functions, are part of the cost of their labor.

This view aligns with how industrial civilizations think about economic compensation. Companies do not pay employees only for the calories they burn typing; they pay them a salary that supports their entire life (during work hours at least, and possibly even a living wage). Environmental accounting should follow the same principle.

This approach is reasonable when comparing human labor to LLMs. While LLMs have quantifiable energy and resource costs, human labor involves interconnected environmental impacts beyond immediate tasks. Considering total environmental impact during work hours provides a more accurate representation of human work costs compared to LLM alternatives^41^.

Our approach aims to reframe the discussion of environmental impacts in labor and production. Instead of allowing corporations to externalize costs by focusing only on task-specific impacts, we argue for a view that acknowledges the full scope of resources dedicated to work activities. This invites deeper consideration of environmental trade-offs in various production modes, including potential substitution of human labor with LLMs.

### Sustainability and economic implications

At first glance, this study’s findings suggest that replacing human labor with AI could lead to substantial environmental benefits, as the direct environmental footprint of LLMs is significantly lower than that of humans for the same output. The comparative LCA results highlight the substantial environmental and economic advantages of Llama-3-70B over human labor in content creation. Across all four metrics—energy consumption, water consumption, carbon emissions, and costs—Llama-3-70B outperforms human labor by orders of magnitude, with human-to-LLM ratios ranging from 40 to 150. When compared to a lightweight AI model (Gemma-2B-it), the ratios range from 1200 to 4400. For the case of India, the human-to-AI ratios are between 3.4 and 16 for a typical LLM and between 130 and 1100 for a lightweight LLM. These findings emphasize the potential of LLMs to reduce the environmental impact of knowledge work and creative tasks, while simultaneously reducing costs. Despite our conservative comparison (e.g., using lower energy and cost values for human labor when applicable), however, we should interpret this study’s findings with cautious optimism. As model sizes continue growing (e.g., recently released Llama-3.1-405B^16^), the energy consumption of LLMs as well as the environmental footprint will likely increase substantially. As a consequence, LLMs may be more energy-consuming than human labor, especially for Indian residents. Thus, we emphasize the need for ongoing research efforts to ensure the energy efficiency and sustainability of LLMs in the long term.

The economic effects of LLM adoption extend beyond the immediate environmental benefits shown in our analysis. While LLMs can reduce the environmental impact of content creation compared to human labor, a straightforward replacement is improbable. LLM integration into various industries will likely be influenced by factors such as the rebound effect and profit-seeking behavior. Moreover, the current pricing provided by OpenAI may be heavily subsidized in order to drive continued user growth. As a result, the economic cost of LLMs could rise in the future.

LLM adoption will likely have immediate, mid-term, and long-term consequences for the economy and society. The initial impact will likely be increased productivity in content creation. Writers, illustrators, and other creators can use LLMs to work more efficiently and potentially produce better output by exploring more possibilities in their creative process. This could increase content volume and variety, possibly benefiting consumers through lower prices and more choices. However, it might also decrease demand for traditional content creation jobs.

As these changes occur, the job market will likely shift. While some traditional content creation roles may diminish, new opportunities will likely appear. These could include content creators skilled in using LLMs, who might earn higher wages, as well as supervisory roles like editors, curators, and LLM system managers. There will also be a need for technicians to maintain and improve LLM systems. This shift will require changes in education and job training to develop new skills. The lower costs and easier entry into content creation could also encourage new business models and increase competition in content-focused industries.

The long-term effects of widespread LLM adoption could be significant and may take years to become apparent. Industries that rely heavily on content creation may need to change how they operate. There is a risk of growing inequality between those who can use AI technologies effectively and those who cannot. How we view creativity, originality, and the nature of work may change as AI-generated content becomes common. We might see a split in the content market: high-volume, low-cost AI content alongside more expensive human-created content. LLMs are already affecting copyright laws, which could change how we value and protect intellectual property.

To illustrate potential economic impacts, we can consider a hypothetical scenario where LLMs are adopted for a significant portion of content creation tasks in the U.S. over the next decade. Assuming current minimum wage rates and LLM costs, this could result in substantial direct cost savings in labor costs for content creation. We might see significant job market shifts, with potential displacement of many content creation jobs, partially offset by the creation of new roles in LLM management and specialized content creation. Notable productivity gains could emerge, with a potential multiple-fold increase in content output per dollar spent. Additionally, we might observe market expansion in content-related industries due to reduced costs and increased accessibility.

These potential outcomes highlight the need for careful planning and proactive policies as LLM use increases. While the immediate benefits in resource efficiency and cost savings are clear, the broader effects on society, the economy, and the environment are varied and interconnected. The actual path of LLM integration will likely involve both human and LLM-driven work, rather than LLMs simply replacing humans. How LLM capabilities, human skills, market needs, and consumer preferences interact will shape the future of content creation and distribution in ways we can not fully predict.

As this transition occurs, more research is needed to measure these impacts accurately and develop ways to reduce potential negative consequences while maximizing the benefits of this technology. The main challenge is how to use the environmental and productivity advantages of LLMs while ensuring fair economic outcomes and maintaining the value of human creativity and expertise.

### Ethical considerations and challenges

Furthermore, while LLMs like Llama-3-70B have demonstrated impressive language generation capabilities, they are also known to produce biased, inconsistent, or factually incorrect outputs^42,43^. (Whether LLMs are more biased, etc., than typical humans, though, is an open question.) The widespread use of LLMs for content creation may exacerbate the spread of misinformation, perpetuate societal biases, or lead to the erosion of trust in online content, or conversely, reduce those effects by replacing even more flawed humans. Addressing these challenges will require the development of robust quality control mechanisms, fact-checking processes, and ethical guidelines for the use of LLMs in content creation^44^.

The potentially substantial environmental benefits of LLMs over human labor for content creation tasks present a complex ethical landscape that merits careful consideration. On one hand, the dramatic reduction in energy consumption, water usage, and carbon emissions offered by LLMs aligns with urgent global sustainability goals and could contribute significantly to mitigating climate change. This environmental advantage creates a strong ethical argument for their widespread adoption.

On the other hand, the potential societal impacts of LLMs-including job displacement, the spread of misinformation, and the perpetuation of biases-raise equally important ethical concerns. The tension between these competing ethical considerations highlights the need for a nuanced approach to LLM implementation. It may be necessary to develop frameworks that balance the environmental benefits of LLMs with strategies to mitigate their potential negative societal impacts. This could include investing in retraining programs for displaced workers, implementing strict content verification processes, and continuously refining LLMs to reduce biases.

Ultimately, the ethical deployment of LLMs will require ongoing attention to ensure that the pursuit of environmental sustainability through LLMs does not come at the cost of social equity and information integrity. It is also equally important to strengthen research efforts to ensure the long-term sustainability of LLMs, especially as model sizes and energy consumption of LLMs continue to increase.

### Limitations and future research directions

This study has several limitations that we address here. These limitations also point toward opportunities for future research. First, the environmental and economic impacts of Llama-3-70B/Gemma-2B-it and human labor may vary depending on the nature of the content creation task. Our analysis focused on a relatively simple task of writing a 500-word page of content, and the results may not be generalizable to more complex or domain-specific tasks.

Second, our study relied on publicly available data and assumptions about the energy consumption, water consumption, carbon emissions, and economic costs of Llama-3-70B/Gemma-2B-it and human labor. While we have made efforts to use the most reliable and up-to-date data sources available, there may be uncertainties and variations in the actual impacts based on the specific hardware and infrastructure used, the geographic location, and the individual behavior of human workers. For example, where the human worker lives makes a large difference in their impact per unit of work produced^10^.

Third, our LCA approach does not account for the potential long-term environmental and economic impacts of LLM adoption, such as the effects on job displacement, skills development, and innovation. Future research should seek to address these concerns.

Finally, our study compared Llama-3-70B/Gemma-2B-it to human labor for content creation, but there are other LLMs and AI-based content creation tools available, each with their own environmental and economic impacts. In future research, we would like to include a broader range of LLMs and content creation approaches, as well as explore the potential for combining human and AI capabilities for optimal performance and sustainability.

As the U.S. National Academies wrote in their 2012 report, Computing Research for Sustainability, “sustainability is not, at its root, a technical problem, nor will merely technical solutions be sufficient. Instead, deep economic, political, and cultural adjustments will ultimately be required, along with a major, long-term commitment in each sphere to deploy the requisite technical solutions at scale. Nevertheless, technological advances and enablers have a clear role in supporting such change^45^.” Our findings demonstrate that LLMs can significantly reduce the environmental footprint of content creation in comparison to human labor, highlighting the potential of this technology to contribute to sustainability efforts in the realm of work. However, the actual impact of LLMs on sustainability will depend on a range of cultural, social, and economic factors that shape their development and deployment, which could lead to either a net reduction or increase in environmental impact. We present the analyses described below as a step toward broader understanding of the role of LLMs in the future of sustainable work.

## Methods

We conducted a comparative life cycle assessment (LCA) of the environmental and economic costs of AI and human labor for the task of writing a 500-word page of content. We considered two different scenarios: typical LLM vs. human and lightweight LLM vs. human. We used Meta’s Llama-3-70B as a representative example of typical LLMs, and Gemma-2B-it as an example of lightweight LLMs. Focusing on the U.S., our analysis quantified and compared the energy consumption, water consumption, carbon emissions, and economic costs associated with each approach. Unless otherwise specified, all the estimates of human-to-LLM ratios were rounded to have two significant figures.

It is important to note that our analysis focuses on these specific models and this particular task as illustrative examples rather than as a comprehensive study. We acknowledge that there are countless LLMs available, each with varying capabilities and environmental impacts, and that content creation encompasses a wide range of tasks beyond the 500-word page we consider here. Our aim is to provide a methodological framework and initial insights that can be extended to other models and tasks in future research.

We focused on text generation as a concrete example application and provided the details of estimating the environmental and economic costs of LLM vs. humans. This approach allows us to investigate a specific use case while establishing a methodology that can be applied more broadly in future studies.

- *Overview of LCA.* LCA is a standardized methodology (ISO 14040/14044) for assessing the environmental impacts of various products and services across their life cycle^13,14^. Our LCA followed the four stages described in the ISO standards:
  - Goal and Scope Definition: We defined the goal of the study as comparing the environmental and economic impacts of Llama-3-70B and human labor for content creation. The functional unit was set as one 500-word page of content, and the system boundaries included energy consumption, water consumption, carbon emissions, and economic costs associated with each approach. To reflect recent improvements in efficiency, we also considered Gemma-2B-it, a lightweight open LLM^40^.
  - Life Cycle Inventory (LCI) Analysis: We collected data relating to both inputs and outputs of the two approaches to content creation under analysis: Llama-3-70B and human labor. For Llama-3-70B, we used publicly available data on the model’s energy consumption^15^ and adjusted for the 500-word writing task, and calculated its environmental footprints accordingly^46,47^. For Gemma-2B-it, we measured its energy consumption by running it on Google Colab equipped with Nvidia A100 and calculated the environmental footprints by assuming that it was deployed in the same environment as Llama-3-70B. For human labor, we used amortized energy consumption and environmental footprints based on the U.S. per capita average. To favor the calculation for humans and avoid potential double counting, we excluded the energy and environmental footprints of devices used by humans for performing the task.
  - Life Cycle Impact Assessment (LCIA): We calculated the environmental impacts of AI and human labor based on the LCI data. Wherever applicable, this included the direct and indirect energy consumption, water consumption, and carbon emissions that result from each approach, as well as the economic costs based on the pricing of OpenAI’s API, the U.S. federal minimum wage and India’s national minimum wage (excluding any applicable fringe benefits)^37–39^.
  - Interpretation: We analyzed the results of the LCIA and compared the environmental and economic impacts of Llama-3-70B and Gemma-2B-it vs. human labor for content creation.

## Additional information

Portions of this article were drafted and/or revised in collaboration with Anthropic’s Claude LLM system, following best practices^48–50^. All content was reviewed and verified by the research team.

## Data Availability

All the data supporting the results reported in the article can be found in our texts and references.

## References

[1] Wang, Y., Pan, Y., Yan, M., Su, Z. & Luan, T. H. A survey on ChatGPT: AI-generated contents, challenges, and solutions. *IEEE Open J. Comput. Soc.* **4**, 280–302. 10.1109/OJCS.2023.3300321 (2023).

[2] Sheth, A., Yip, H. Y., Iyengar, A. & Tepper, P. Cognitive services and intelligent chatbots: Current perspectives and special issue introduction. *IEEE Internet Comput.* **23**, 6–12. 10.1109/MIC.2018.2889231 (2019). 33746506 10.1109/MIC.2018.2889231 PMC7971175

[3] Zeng, Z. et al. An extensive study on pre-trained models for program understanding and generation. In Proceedings of the 31st ACM SIGSOFT International Symposium on Software Testing and Analysis, ISSTA 2022, 39–51, 10.1145/3533767.3534390 (Association for Computing Machinery, New York, NY, USA, 2022).

[4] Stella, F., Della Santina, C. & Hughes, J. How can LLMs transform the robotic design process?. *Nat. Mach. Intell.* **5**, 561–564. 10.1038/s42256-023-00669-7 (2023).

[5] Schwartz, R., Dodge, J., Smith, N. A. & Etzioni, O. Green AI. *Commun. ACM* **63**, 54–63. 10.1145/3381831 (2020).

[6] Dhar, P. The carbon impact of artificial intelligence. *Nat. Mach. Intell.* **2**, 423–425. 10.1038/s42256-020-0219-9 (2020).

[7] Strubell, E., Ganesh, A. & McCallum, A. Energy and policy considerations for modern deep learning research. *Proc. AAAI Conf. Artif. Intell.* **34**, 13693–13696. 10.1609/aaai.v34i09.7123 (2020).

[8] Li, P., Yang, J., Islam, M. A. & Ren, S. Making AI less “thirsty”’: Uncovering and addressing the secret water footprint of AI models. Communications of the ACM (to appear) (2024).

[9] IEA. Electricity 2024: Analysis and forecast to 2026. IEA Report (2024, https://www.iea.org/reports/electricity-2024).

[10] Tomlinson, B., Black, R. W., Patterson, D. J. & Torrance, A. W. The carbon emissions of writing and illustrating are lower for ai than for humans. *Sci. Rep.* **14**, 3732 (2024). 38355820 10.1038/s41598-024-54271-x PMC10867074

[11] Henderson, P. et al. Towards the systematic reporting of the energy and carbon footprints of machine learning. *J. Mach. Learn. Res.* **21** (2020).

[12] Kaack, L. H. et al. Aligning artificial intelligence with climate change mitigation. *Nat. Clim. Chang.* **12**, 518–527 (2022).

[13] ISO 14040:2006. Environmental management - life cycle assessment - principles and framework. International Organization for Standardization, Geneva, Switzerland (2006).

[14] ISO 14044:2006. Environmental management - life cycle assessment - requirements and guidelines. International Organization for Standardization, Geneva, Switzerland (2006).

[15] Dubey, A. & Others. The llama 3 herd of models (2024). 2407.21783.

[16] Meta. Introducing Llama 3.1: Our most capable models to date. https://ai.meta.com/blog/meta-llama-3-1/.

[17] Stojkovic, J., Zhang, C., Íñigo Goiri, Torrellas, J. & Choukse, E. DynamoLLM: Designing LLM inference clusters for performance and energy efficiency (2024). 2408.00741.

[18] Patel, P. et al. Characterizing power management opportunities for llms in the cloud. In Proceedings of the 29th ACM International Conference on Architectural Support for Programming Languages and Operating Systems, Volume 3, ASPLOS ’24, 207–222, 10.1145/3620666.3651329 (Association for Computing Machinery, New York, NY, USA, 2024).

[19] U.S. Energy Information Administration. Frequently asked questions: How much carbon dioxide is produced per kilowatthour of U.S. electricity generation? https://www.eia.gov/tools/faqs/faq.php?id=74&t=11.

[20] Microsoft. How Microsoft measures datacenter water and energy use to improve Azure Cloud sustainability. Microsoft Azure Blog (2022).

[21] Reig, P., Luo, T., Christensen, E. & Sinistore, J. Guidance for calculating water use embedded in purchased electricity. World Resources Institute (2020).

[22] Luccioni, A. S., Viguier, S. & Ligozat, A.-L. Estimating the carbon footprint of BLOOM, a 176B parameter language model. *J. Mach. Learn. Res.* **24** (2024).

[23] Gupta, U. et al. Act: designing sustainable computer systems with an architectural carbon modeling tool. In Proceedings of the 49th Annual International Symposium on Computer Architecture, ISCA ’22, 784–799, 10.1145/3470496.3527408 (Association for Computing Machinery, New York, NY, USA, 2022).

[24] Nguyen, S., Zhou, B. & Liu, Y. D. S. Towards sustainable large language model serving. In HotCarbon (2024).

[25] Singh, S. Introducing Meta Llama 3: The most capable openly available LLM to date. https://ai.meta.com/blog/meta-llama-3/ (2024).

[26] Patel, P. et al. Splitwise: Efficient generative LLM inference using phase splitting. In 2024 ACM/IEEE 51st Annual International Symposium on Computer Architecture (ISCA), 118–132, 10.1109/ISCA59077.2024.00019 (2024).

[27] Microsoft. Environmental sustainability report. https://query.prod.cms.rt.microsoft.com/cms/api/am/binary/RW1lhhu (2024).

[28] U.S. Energy Information Administration. Per capita U.S. residential electricity use was flat in 2020, but varied by state. https://www.eia.gov/todayinenergy/detail.php?id=49036.

[29] U.S. Energy Information Administration. Frequently asked questions: How much energy does a person use in a year? https://www.eia.gov/tools/faqs/faq.php?id=85&t=1.

[30] U.S. Energy Information Administration. U.S. energy-related carbon dioxide emissions, 2023. https://www.eia.gov/environment/emissions/carbon/.

[31] Climate Transparency. Climate transparency report India. https://www.climate-transparency.org/wp-content/uploads/2022/10/CT2022-India-Web.pdf (2022).

[32] Google. Environmental report. https://www.gstatic.com/gumdrop/sustainability/google-2023-environmental-report.pdf (2023).

[33] U.S. Environmental Protection Agency. Water stats. https://www.epa.gov/watersense/statistics-and-facts. 11645578

[34] The World Bank. Indicator name annual freshwater withdrawals, domestic (% of total freshwater withdrawal, ER.H2O.FWDM.ZS). https://databank.worldbank.org/metadataglossary/world-development-indicators/series/ER.H2O.FWDM.ZS.

[35] U.S. Census Bureau. Nation’s urban and rural populations shift following 2020 census. https://www.census.gov/newsroom/press-releases/2022/urban-rural-populations.html.

[36] Indian Ministry of Jal Shakti. Per capita availability of water. https://pib.gov.in/PressReleasePage.aspx?PRID=1604871.

[37] OpenAI. OpenAI API Pricing. https://openai.com/api/pricing/.

[38] U.S. Department of Labor. Minimum wage. https://www.dol.gov/general/topic/wages/minimumwage (2023).

[39] Government of India Chief Labour Commissioner (Central). Minimum wages. https://clc.gov.in/clc/min-wages.

[40] Google. Gemma: Introducing new state-of-the-art open models. https://blog.google/technology/developers/gemma-open-models/ (2024).

[41] Tomlinson, B., Black, R. W., Patterson, D. J. & Torrance, A. W. The carbon emissions of writing and illustrating are lower for AI than for humans. *Sci. Rep.* **14** (2024). 10.1038/s41598-024-54271-x PMC10867074 38355820

[42] Bender, E. M., Gebru, T., McMillan-Major, A. & Shmitchell, S. On the dangers of stochastic parrots: Can language models be too big? In Proceedings of the 2021 ACM Conference on Fairness, Accountability, and Transparency, FAccT ’21, 610–623, 10.1145/3442188.3445922 (Association for Computing Machinery, New York, NY, USA, 2021).

[43] McGuffie, K. & Newhouse, A. The radicalization risks of GPT-3 and advanced neural language models. ArXiv **abs/2009.06807** (2020).

[44] Creel, K. & Hellman, D. The algorithmic leviathan: Arbitrariness, fairness, and opportunity in algorithmic decision-making systems. *Can. J. Philos.* **52**, 26–43. 10.1017/can.2022.3 (2022).

[45] Estrin, D. L. & Millett, L. I. Computing Research for Sustainability (National Academies Press, 2012).

[46] Gupta, U. et al. Chasing carbon: The elusive environmental footprint of computing. In 2021 IEEE International Symposium on High-Performance Computer Architecture (HPCA), 854–867, 10.1109/HPCA51647.2021.00076 (2021).

[47] Bashroush, R. & Lawrence, A. *Tackling IT’s wasted terawatts* (Uptime Institute, Beyond PUE, 2020).

[48] Tomlinson, B., Torrance, A. W. & Black, R. W. ChatGPT and works scholarly: Best practices and legal pitfalls in writing with AI. *SMU Law Rev. Forum* **76**, 108 (2023).

[49] Editorials, Nature. Tools such as ChatGPT threaten transparent science; here are our ground rules for their use. *Nature* **613**, 10–1038 (2023). 10.1038/d41586-023-00191-1 36694020

[50] Nature Machine Intelligence. Writing the rules in AI-assisted writing. *Nat. Mach. Intell.* **5**, 469. 10.1038/s42256-023-00678-6 (2023).

